# Implementation of a point-of-care ultrasound archiving system and governance framework in a UK physician-paramedic staffed helicopter emergency medical service

**DOI:** 10.1186/s13049-024-01224-y

**Published:** 2024-06-03

**Authors:** Shadman Aziz, Christopher T. Edmunds, Jon Barratt

**Affiliations:** 1 Department of Research, Audit, Innovation, and Development (RAID), East Anglian Air Ambulance, Norwich, UK; 2 Emergency and Critical Care Departments, North West Anglia Foundation Trust, Peterborough, UK; 3 University of East Anglia, Norwich, UK; 4 Academic Department of Military Emergency Medicine, Royal Centre for Defence Medicine (Research & Clinical Innovation), Birmingham, UK; 5 Emergency Department, University Hospitals of North Midlands NHS Trust, Stoke-on-Trent, UK

**Keywords:** Prehospital, HEMS, Point-of-care ultrasound, Governance, Image archiving

## Abstract

**Introduction:**

There has been a rapid expansion in the use of point-of-care ultrasonography (POCUS) by emergency medical services (EMS). However, less than a third of UK EMS utilise imaging archiving for POCUS, and fewer review saved images as part of a clinical governance structure. This paper describes the implementation of a novel image archiving system and a robust clinical governance framework in our UK physician-paramedic staffed helicopter emergency medical service (HEMS).

**Methods:**

A retrospective database review was conducted of all patients attended by East Anglian Air Ambulance (EAAA) between the introduction of a new POCUS device and image archiving system on 1 December 2020 to 31 January 2024. All patients with recorded POCUS examinations were included. Images from POCUS examinations at EAAA are archived on a cloud-based server, and retrospectively reviewed within 24 h by an EAAA POCUS supervisor. Image quality is graded using a 5-point Likert-type scale, agreement between reviewer and clinician is recorded and feedback is provided on scanning technique. T-tests were used to assess the difference in image quality between physicians and paramedics. Inter-rater reliability between reviewers and clinicians was assessed using Cohen’s kappa (κ).

**Results:**

During the study period, 5913 patients were attended by EAAA. Of these, 1097 patients had POCUS images recorded. The prevalence of POCUS during the study period was 18.6%. 1061 patient examinations underwent quality assurance (96.7%). The most common POCUS examination was echocardiography (60%), predominantly during cardiac arrest. The primary scanning clinician was a paramedic in 25.4% of POCUS examinations. Across all examination types; image quality was not significantly different between physicians and paramedics and agreement between reviewers and clinicians was strong (κ > 0.85).

**Conclusions:**

In this service evaluation study, we have described outcomes following the introduction of a new POCUS device, image archiving system and governance framework in our HEMS. Paramedics were the primary scanning clinician in a quarter of scans, with image quality comparable to physicians. Almost all scans underwent quality assurance and inter-rater reliability was strong between clinicians and reviewers. Further research is required to investigate the diagnostic accuracy of POCUS and to demonstrate the effect of utilising prehospital POCUS to refine diagnosis on clinical outcomes.

## Background

There has been a rapid expansion in the use of point-of-care ultrasonography (POCUS) by UK prehospital emergency medical services (EMS) [1]. Prehospital POCUS is feasible and has been shown to enhance clinical assessment and diagnosis, aid decision making, and facilitate ultrasound-guided interventions [1–4]. However, the reported diagnostic accuracy of prehospital POCUS is variable, and there is a paucity of literature on the quality of prehospital POCUS examinations [5, 6]. This is partially due to the lack of image archiving, which precludes retrospective review of scans and quality assurance (QA) processes.

Guidance published in 2021 by the British Medical Ultrasound Society (BMUS) has recommended that all POCUS examinations conducted outside of radiology departments should be subject to robust clinical governance processes, and that images should be permanently archived for medicolegal purposes [7]. This guidance was expanded by the BMUS and the Royal College of Radiologists (RCR) in 2023 to include recommendations for regular audit of recorded scans to perform QA and facilitate learning [8]. Despite this, less than a third of UK EMS utilise imaging archiving for POCUS, and fewer review saved images for clinical governance [1].

In 2019, a dedicated programme was started at East Anglian Air Ambulance (EAAA) to improve POCUS delivery by the service. This article details the establishment of a new POCUS device, including the implementation of an image archiving system and a robust clinical governance framework in our UK helicopter emergency medical service (HEMS). We also report our initial experience and outcomes from the new QA process.

## Methods

### Study setting

EAAA is a UK HEMS that provides prehospital critical care to the statutory ambulance service in the East of England (East of England Ambulance Service NHS Trust (EEAST)). The East of England is a geographic area of 20,000 km^2^ and has approximately 6.4 million inhabitants [9]. The HEMS system consists of two teams each composed of at least one physician and a critical care paramedic. Teams are dispatched from one of two bases (Cambridge and Norwich) by either helicopter or ground response vehicle, depending on patient location, weather, and time of day [10, 11]. Each year EAAA is tasked to approximately 3000 primary missions and attends 2000 patients. Approximately 30% of cases attended by EAAA are out-of-hospital cardiac arrests (OHCA); 25% road traffic accidents; 25% other traumatic incidents; and 20% other medical emergencies.

### Prehospital ultrasound at EAAA

POCUS has been utilised at EAAA since 2015. Clinical cases were already subjected to routine detailed review, but with an increase in the use of POCUS, there was an acknowledgement that it was essential to develop a robust education and governance framework to ensure accurate image interpretation and safe clinical application of POCUS. The POCUS hardware and software in use in 2015 had lengthy workflows for image archiving, which limited the service’s ability to retrospectively review images. In 2019, a management of change process was started at EAAA to improve POCUS delivery by the service. This coincided with developments in ultrasound technology that allowed for easier image archiving and remote review of POCUS studies using cloud-based storage, which provided an opportunity to improve engagement with clinical governance processes.

It was recognised that POCUS skillsets and accreditation vary depending on the clinician’s professional background, experience, and base specialty (if a physician). It was agreed by the POCUS working group that the Royal College of Emergency Medicine (RCEM) Core Level 1 suite of POCUS examinations provided utility and coverage of the full spectrum of incidents attended by EAAA [12]. Accordingly, the following examinations constitute the minimum expected standard for clinicians working at EAAA:

- Cardiac ultrasound
  - Echocardiography in life support (ELS) – Echocardiography performed during cardiac arrest to identify reversible causes of arrest (e.g., cardiac tamponade) or to differentiate between low-flow contractile states and cardiac standstill
  - If indicated, other detailed echocardiography examinations can be performed by clinicians accredited via alternative pathways (e.g., Focused Ultrasound in Intensive Care, British Society of Echocardiography)
- Focused assessment with sonography for trauma (FAST) / Extended focused assessment with sonography for trauma (E-FAST)
  - Abdominal sonography to identify free peritoneal fluid in the right upper quadrant, left upper quadrant and pelvis (FAST)
  - FAST examination with additional lung views to identify the presence of pneumothorax and/or haemothorax (E-FAST)
- Lung ultrasound
  - Lung sonography used in isolation to identify the presence of pneumothorax and/or haemothorax
- Assessment of abdominal aortic aneurysms (AAA)
  - Sonography to identify the presence of a dilated or ruptured aortic aneurysm where the clinical history is of concern
- Ultrasound-guided vascular access (USG-VA)
  - Ultrasound-guided venous (peripheral or central) and arterial access

### Education and competency

**Fig. 1:**
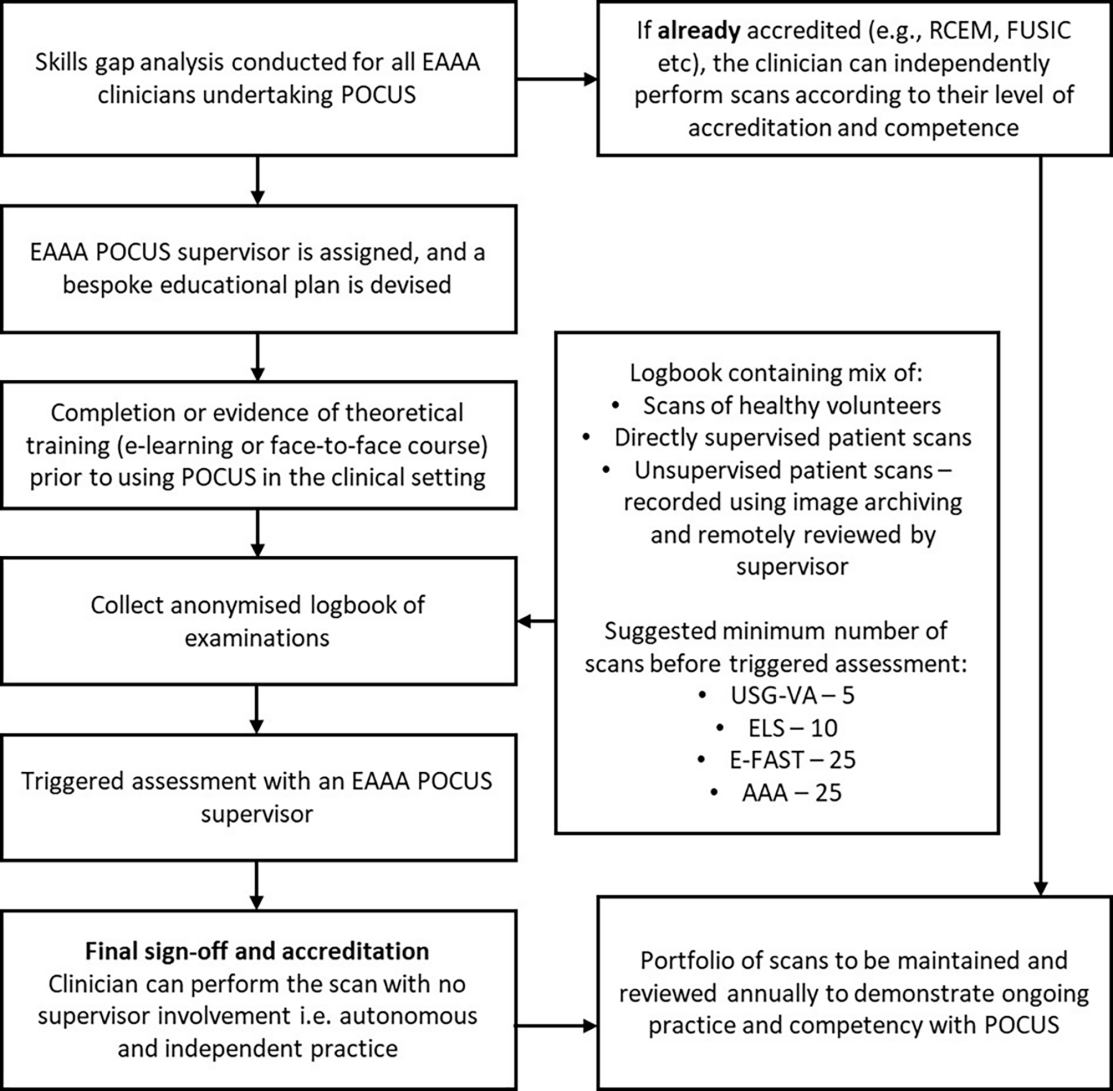
Current EAAA Level 1 ultrasound sign off process. Abbreviations: AAA: Assessment of abdominal aorta; EAAA: East Anglian Air Ambulance, E-FAST: Extended focused abdominal sonography in trauma; ELS: Echocardiography in life support; FUSIC: Focused Ultrasound in Intensive Care; POCUS: Point-of-care ultrasound; USG-VA: Ultrasound-guided vascular access

The development of an educational programme was crucial to support the improvement of POCUS governance processes within EAAA. A new sign-off process was developed to support clinicians working towards the equivalent of RCEM Level 1 accreditation. Each clinician is allocated a POCUS mentor to understand prior clinical exposure to POCUS and to identify specific learning needs. In addition, a one-day POCUS training course was designed to cover the theoretical and practical elements of prehospital POCUS. The course consists of lectures covering the basic science of ultrasound and each modality used with EAAA. This is supplemented with talks covering the latest evidence and the modifications of POCUS required for the prehospital environment. Theory is then consolidated in a series of small-group workshops with live volunteers to focus on scanning technique; and moulages with application of POCUS in prehospital scenarios. Internal candidates are enrolled onto the course to ensure the basic skills of POCUS are taught to all clinical team members. The course is now endorsed by the University of East Anglia, runs twice a year and has been opened to external candidates seeking to develop their prehospital POCUS capabilities. The EAAA POCUS sign-off process is summarised in Fig. 1.

Although there are suggested minimum numbers of scans prior to sign off, this is used as a guide. POCUS is considered an ‘Entrustable professional activity’, so the final sign-off evaluates the clinician’s ability to perform POCUS without direct supervision. Recognition of each individuals’ speed of skill acquisition is crucial, and the final sign off is focused on image acquisition technique, whilst considering the clinical context and the limitations of prehospital POCUS. One year after completing the initial training course, EAAA clinicians also have the opportunity to consolidate their skills in a bespoke ‘refresher’ course, which provides a safe environment to address individual educational needs and focus on specific skills.

POCUS examinations are only conducted if clinically indicated to ensure they do not distract from the patient’s immediate clinical needs or unnecessarily prolong scene times. Findings are only used to influence patient management if the scanning clinician is fully signed-off or if an accredited supervisor is present as part of the clinical team. Unsupervised patient scans are encouraged to build the clinician’s logbook and experience with POCUS but are not used to influence patient management. Clinicians are also able to perform POCUS examinations independently if accredited via alternative pathways (e.g., Focused Ultrasound in Intensive Care, Focused Acute Medicine Ultrasound etc.), once they are familiar with the EAAA usage protocols.

### Ultrasound device and workflow

**Fig. 2:**
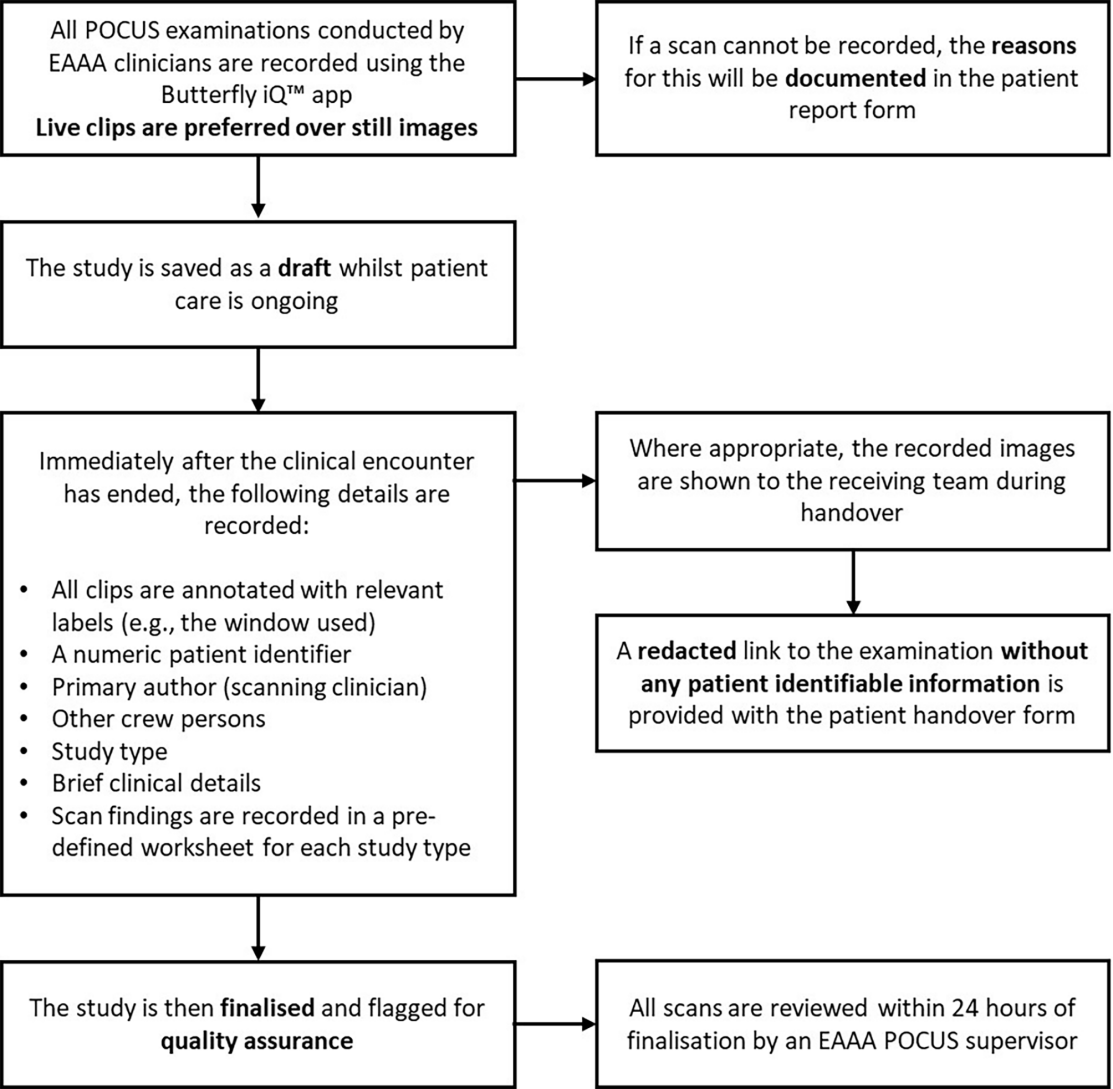
Current EAAA POCUS workflow. Abbreviations: East Anglian Air Ambulance, POCUS: Point-of-care ultrasound

After careful consideration of a number of contemporary POCUS devices, the Butterfly iQ™ (Butterfly Network, Massachusetts, USA) was selected for the EAAA POCUS programme. This device has several advantages that help facilitate a POCUS programme. Principally, it utilises a novel silicone-chip based technology in a single, portable, battery-operated probe; obviating the need for multiple transducers. This is advantagenous in a prehospital HEMS service where space for equipment is limited. Additionally, POCUS images and short video clips can be recorded and stored in a secure cloud-based data environment that is compliant with General Data Protection Regulation (EU 2016/679) and UK Data Protect Act (2018) legislation. This allows for image achiving, retrospective review of images, logbook collection, clinical governance and QA. POCUS using the Butterfly iQ™ was formally introduced at EAAA on 1 December 2020. The EAAA POCUS workflow is summarised in Fig. 2.

Documentation includes all the minimum elements as set out by the BMUS guidelines for specialists practicing ultrasound independently of radiology departments [8]. POCUS findings were initially documented freehand in the patient record. However, it has been demonstrated that POCUS documentation quality is improved by the use of proformas and it was recognised that freehand documentation limited the ability for data analysis [13]. As a result, predefined worksheets for each POCUS examination were introduced in November 2022 to standardise documentation of findings.

The primary author of the documentation is defined as the clinician who principally conducted the scan (i.e., held the probe). However, the delivery of prehospital critical care as a team may mean that there is crossover and influence of the scan by more experienced members of the clinical team, particularly if the scanning clinician is actively being supervised on scene. Therefore, other team members are recorded as secondary/tertiary authors on the cloud-based server and QA feedback is sent to the entire team.

### Clinical governance

All recorded POCUS examinations are reviewed by an EAAA POCUS supervisor within 24 h of finalisation. Where the findings of the reviewer differ from the scanning clinician, this is escalated to the EAAA POCUS lead to provide a consensus. If it is felt that the POCUS had been incorrectly interpreted, and if this may affect patient care, the receiving team are contacted to discuss the interpreted findings and ensure appropriate clinical management is undertaken. If the patient is not in a clinical setting and has been discharged from care, they are contacted directly and a letter to both the patient and GP is sent with suggested follow up.

QA was initially conducted using a Microsoft PowerApps form and stored on a secure spreadsheet on the EAAA Microsoft SharePoint server. However, this QA workflow was complicated and required multiple time-consuming steps. In order to streamline the QA process, since November 2022, QA is conducted directly using the Butterfly iQ™ image archiving system and stored on their secure cloud-based data environment. QA originally recorded using the Microsoft PowerApps form were retrospectively transferred onto the Butterfly iQ™ system in August 2023.

QA and feedback on image acquisition are recorded using a predefined template (Table 1). Image quality is scored on a five-point Likert-type scale. Examinations are classified as true positive (TP), true negative (TN), false positive (FP) and false negative (FN) based on the reviewer’s interpretation of the scan. Separate comparison to gold-standard imaging findings is conducted if follow-up or radiology reports are available. Feedback is provided on 7 predefined areas of scan technique. In addition, reviewers will comment on whether the scan was ‘clinically indicated’ based on the clinical context described in the patient record; or if further information is required, following discussion with the clinical team. This is done to ensure that clinicians are able to apply POCUS selectively, appropriately, and efficiently in the correct clinical context [14]. Finally, where applicable, written feedback considering the clinician’s individual educational needs is also provided. Automated feedback emails are sent directly to clinicians once the scan has been reviewed (within 24 h). Cases with identified learning points or where POCUS was used to change patient management are flagged for discussion with the wider clinical team in a ‘clinical case review’ meeting. This process ensures continuous institutional learning, improvement, and evolution of the use of POCUS within the service.

**Table 1:**
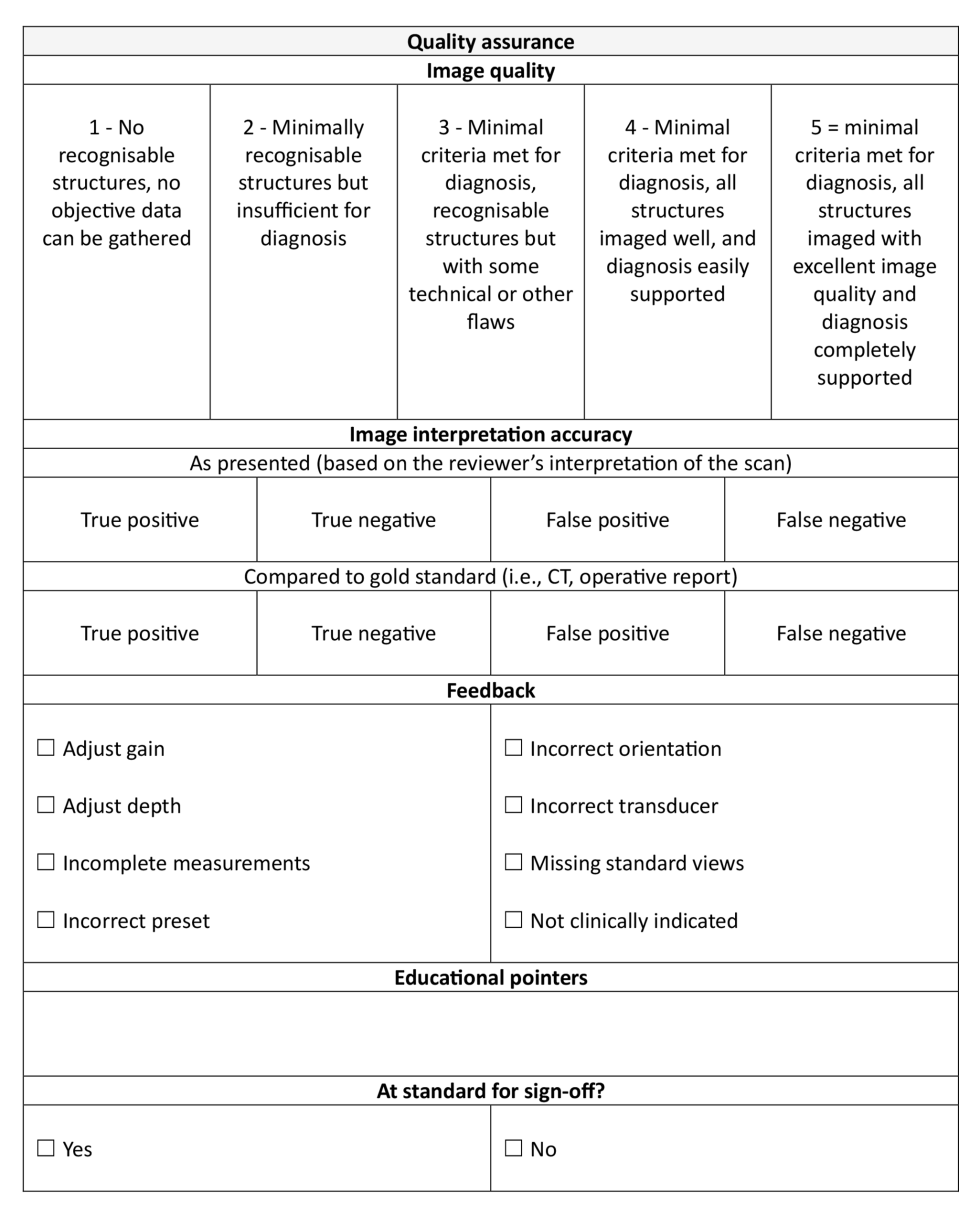
Quality assurance template used at EAAA

### Data collection

A retrospective database review was conducted on a convenience sample of all patients attended by East Anglian Air Ambulance (EAAA) between the introduction of the new POCUS device, image archiving system and governance framework on 1 December 2020 to 31 January 2024 (the date of most recent POCUS QA data).

All patients attended by EAAA who had a POCUS examination and corresponding QA process were included. Pseudonymised QA data from the online image archiving system (Butterfly Network, Massachusetts, USA) were extracted and collated in a single data spreadsheet (Microsoft® Excel® for Microsoft 365, v2308) and stored in a secure data environment. Variables collected were: primary author (scanner) background, scan type, image quality score, feedback on image acquisition technique, and the TP/TN/FP/FN rates as decided by expert reviewers.

### Data analysis

The primary analysis was to report the average quality of images as assessed by expert reviewers and inter-rater reliability (IRR) between the HEMS clinicians conducting the scan and expert reviewers. Manipulation of data and statistical analyses were performed in R statistical programming language (R Foundation for Statistical Computing, Vienna, Austria) [15]. Complete case analysis was utilised for this study. The sample characteristics were described using number (percentage) for categorical variables and mean (± standard deviation (SD)) for continuous variables. T-tests were used to assess differences between means and IRR was assessed using the Cohen’s kappa statistic (κ). A pre-defined significance value of *p* < 0.05 was used throughout.

## Results

During the study period, 5913 patients were attended by EAAA. 1097 patients had POCUS images archived. The prevalence of POCUS during the study period was 18.6%. 1061 patient examinations underwent QA (96.7%). A summary of reviewed scans by examination type is provided in Table 2. Cardiac ultrasound was the most common POCUS examination in this cohort. 25.4% of POCUS examinations were conducted by paramedics.

**Table 2:** Number of reviewed scans organised by examination type and background of the scanner

	All patients *n* = 1061	Paramedic *n* = 269	Physician *n* = 792
Cardiac	633 (60%)	141 (52%)	492 (62%)
FAST / E-FAST	338 (32%)	111 (41%)	227 (29%)
Lung	141 (13%)	32 (12%)	109 (14%)
USG-VA	75 (7.1%)	10 (3.7%)	65 (8.2%)
AAA	24 (2.3%)	4 (1.5%)	20 (2.5%)
Other	22 (2.1%)	9 (2.6%)	15 (1.9%)

Data are expressed as *n=*(%). Abbreviations: AAA: Assessment of abdominal aorta; FAST: Focused assessment with sonography for trauma; E-FAST: Extended FAST; USG-VA: Ultrasound-guided vascular access

**Fig. 3:**
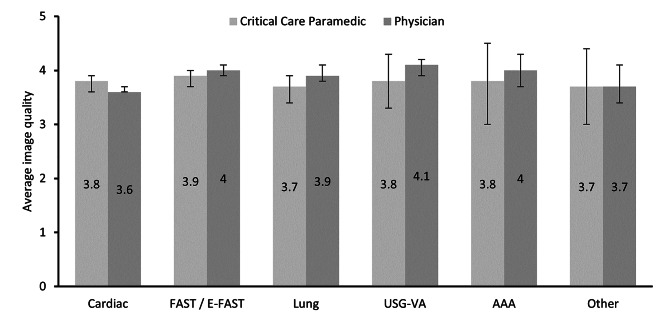
Average image quality organised by examination type and background of the primary scanning clinician. Error bars represent the 95% confidence interval. Abbreviations: AAA: Assessment of abdominal aorta; FAST: Focused assessment with sonography for trauma; E-FAST: Extended FAST; USG-VA: Ultrasound-guided vascular access

Image quality was not significantly different between physicians and paramedics for each examination type (Table 3 and Fig. 3). Image quality was greatest for FAST, USG-VA and AAA scans.

**Table 3:** Image quality organised by examination type and background of the scanner

	Overall	Paramedic	Physician	*P* value^†^
Cardiac	3.7 (0.8)	3.8 (0.7)	3.6 (0.8)	0.1
FAST / E-FAST	4.0 (0.7)	3.9 (0.7)	4.0 (0.7)	0.2
Lung	3.9 (0.7)	3.7 (0.7)	3.9 (0.7)	0.1
USG-VA	4.0 (0.7)	3.8 (0.6)	4.1 (0.7)	0.2
AAA	4.0 (0.6)	3.8 (0.5)	4.0 (0.6)	0.4
Other	3.7 (0.6)	3.7 (0.8)	3.7 (0.6)	0.9

Data are expressed as mean (standard deviation). Abbreviations: AAA: Assessment of abdominal aorta; FAST: Focused assessment with sonography for trauma; E-FAST: Extended FAST; USG-VA: Ultrasound-guided vascular access

^†^Two-sample t-test

Cohen’s kappa calculated between scanner and reviewer showed strong agreement for every diagnostic scan type (Table 4). The highest levels of agreement were found in the small number of ‘other’ scans. Of the core examinations, agreement was highest for lung ultrasound. Cardiac and FAST examinations showed the lowest degree of agreement.

**Table 4:** Image interpretation accuracy organised by diagnostic examination type

	True positives	True negatives	False positives	False negatives	Reviewer agreement with scanner	Kappa
Cardiac	422	161	11	26	94.0%	0.86
FAST / E-FAST	80	233	7	12	94.3%	0.85
Lung	51	83	1	4	96.4%	0.92
AAA	13	10	0	1	95.8%	0.92
Other	11	10	0	0	100.0%	1.00

Data are expressed as *n=*(%) unless otherwise stated. Abbreviations: AAA: Assessment of abdominal aorta; FAST: Focused assessment with sonography for trauma; E-FAST: Extended FAST

**Fig. 4:**
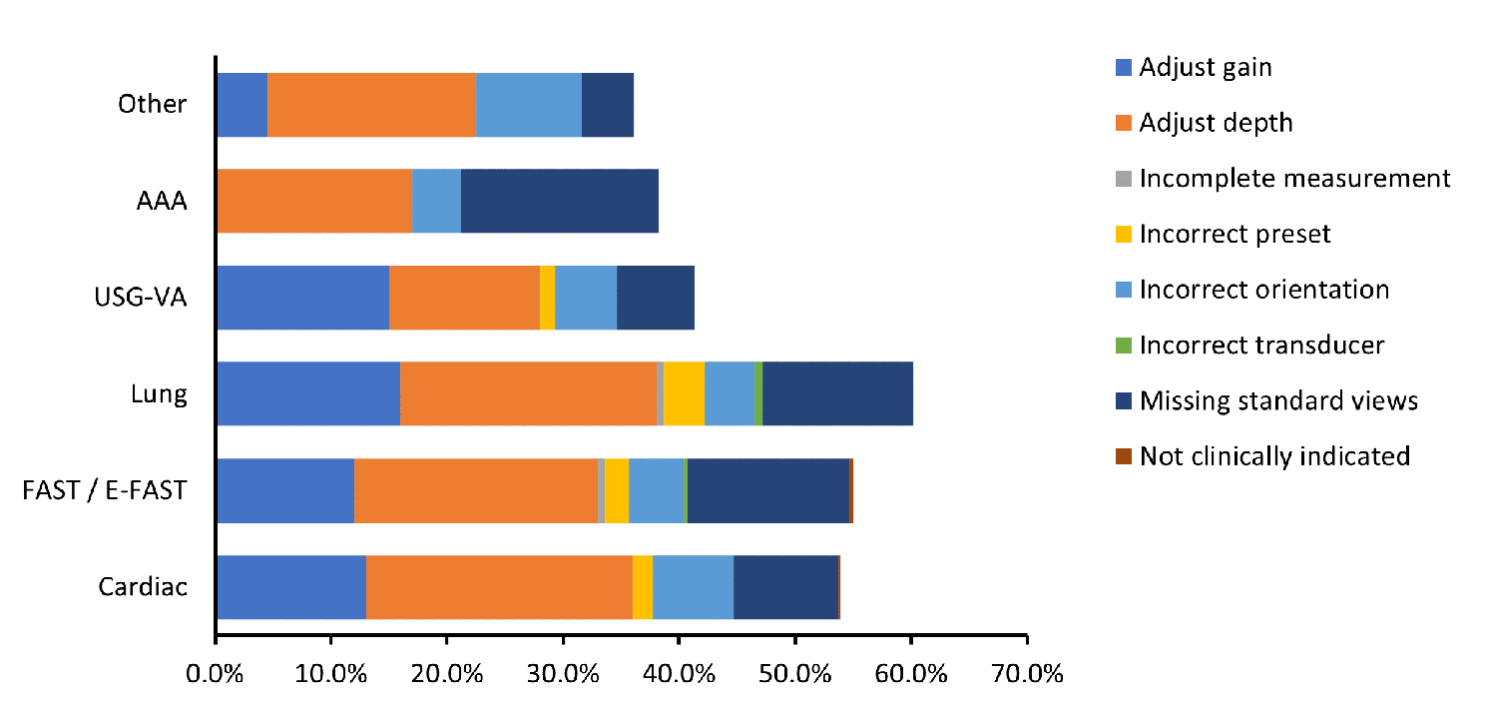
Feedback organised by examination type. Abbreviations: AAA: Assessment of abdominal aorta; FAST: Focused assessment with sonography for trauma; E-FAST: Extended FAST; USG-VA: Ultrasound-guided vascular access

A summary of feedback provided on 7 predefined areas of scan technique and whether the scan was clinically indicated is provided in Table 5. Adjustment of image gain and depth were the most common areas of feedback (Fig. 4).

**Table 5:** Feedback organised by examination type

	Adjust gain	Adjust depth	Incomplete measure-ment	Incorrect preset	Incorrect orientation	Incorrect transducer	Missing standard views	Not clinically indicated
Cardiac	82 (13%)	144 (23%)	0 (0%)	11 (1.7%)	44 (7.0%)	0 (0%)	57 (9.0%)	1 (0.2%)
FAST / E-FAST	40 (12%)	70 (21%)	2 (0.6%)	7 (2.1%)	16 (4.7%)	0 (0%)	46 (14%)	1 (0.3%)
Lung	23 (16%)	31 (22%)	1 (0.7%)	5 (3.5%)	6 (4.3%)	0 (0%)	18 (13%)	0 (0%)
USG-VA	11 (15%)	10 (13%)	0 (0%)	1 (1.3%)	4 (5.3%)	0 (0%)	5 (6.7%)	0 (0%)
AAA	0 (0%)	4 (17%)	0 (0%)	0 (0%)	1 (4.2%)	0 (0%)	4 (17%)	0 (0%)
Other	1 (4.5%)	4 (18%)	0 (0%)	0 (0%)	2 (9.1%)	0 (0%)	1 (4.5%)	0 (0%)

Data are expressed as *n=*(%) unless otherwise stated. Abbreviations: AAA: Assessment of abdominal aorta; FAST: Focused assessment with sonography for trauma; E-FAST: Extended FAST; USG-VA: Ultrasound-guided vascular access

## Discussion

In this retrospective service evaluation study, we have described the introduction of a new POCUS device, image archiving system and governance framework in our HEMS and reported initial results from the QA process. Although there is an absence of specific guidelines around the application of POCUS, five essential pillars have been suggested to ensure the highest standards of clinical quality and safety for any organisation establishing a POCUS programme (governance, infrastructure, administration, education, and quality) [16]. The implementation of POCUS at EAAA followed these key principles, leading to the development of a detailed educational programme to ensure competency of clinicians performing POCUS; a strict workflow utilising mandatory image archiving; and robust QA and clinical governance processes in line with RCR and BMUS guidelines [7, 8]. 

Cardiac ultrasound (including ELS conducted during cardiac arrest) was the most common POCUS examination in our cohort. This aligns with the case-mix attended by EAAA, as OHCA is the most common incident attended by the HEMS team. However, this contrasts with survey data that suggest E-FAST is the most common POCUS study undertaken by HEMS in Europe [3]. Only 2.1% of POCUS examinations were classified as miscellaneous scans, suggesting that the RCEM Level 1 suite of examinations provides good coverage of the diagnostic and procedural indications for POCUS in this UK HEMS service. Although, this may represent the fact that many EAAA clinicians are only trained in this catalogue of examinations, so would not have been able to conduct miscellaneous scans.

In the UK, there is a limited evidence supporting the diagnostic use of prehospital POCUS by non-physicians [17]. In this study, one quarter of scans were conducted by HEMS paramedics, and image quality did not differ significantly when compared to physicians. However, the delivery of prehospital critical care as a team may mean that there is crossover and influence on scan quality by more experienced members of the clinical team, particularly if the scanning clinician is actively being supervised on scene. Due to an inability and inappropriateness to blind other team members to the POCUS study findings, it is not possible to mitigate this in an operational context. Regardless, these data demonstrate that scans conducted by paramedics working in a HEMS system with robust POCUS education and governance can achieve image quality comparable to physicians working in the same service.

Almost all recorded scans underwent QA. This demonstrates that a user-friendly POCUS workflow, coupled with a strong organisational culture of continual learning and improvement with POCUS can achieve high levels of compliance with QA processes. Furthermore, there was strong agreement between reviewers and HEMS clinicians across all examination types, which increases confidence in the POCUS findings. However, due to the belated adoption of predefined worksheets on the cloud-based server in November 2022, it was not possible to download a detailed summary of pathologies found using POCUS over the entire study period. Coupled with the poor unavailability of follow-up data, this study was unable to report the sensitivity and specificity of POCUS examinations compared to definitive in-hospital investigations. Therefore, although inter-rater reliability was strong, this study cannot comment on the diagnostic accuracy of POCUS in our cohort.

Optimisation of image depth and gain were the most common points of feedback highlighted during the QA process, suggesting that these components should be emphasized in the prehospital POCUS curriculum. Depth, gain and the image axis (angle of the ultrasound beam with the structure of interest) are well known determinants of POCUS image quality, the latter of which has been demonstrated to have the steepest learning curve in novice POCUS users [18]. However, although feedback on the image axis is often provided in the written QA feedback, it is currently not one of the 7 predefined areas of scan technique on the electronic QA proforma.

There were no available data on the time taken to perform scans. Therefore, it was not possible to assess the effect of POCUS on prehospital scene times. However, a recent systematic review and meta-analysis found that prehospital FAST scanning reduced the time to diagnosis and intervention, without increasing scene times [19]. In addition, EAAA frequently utilises three-person teams, allowing for concurrent activity, which may actually lead to a reduction in scene times. Furthermore, POCUS can be conducted during transport to hospital, which has no effect on scene times. However, data on the proportion of scans conducted during transit was not available in this study.

### Limitations

This study was retrospective and utilised routinely collected anonymised patient data. QA was conducted by non-radiologist EAAA POCUS supervisors, who are experienced HEMS clinicians and frequently work with the clinical teams conducting the scans. The benefit of using experienced clinicians who understand the operational context is thought to outweigh any potential bias but does introduces a degree of subjectivity when reporting the studies. In order to mitigate this, there are plans to expand the POCUS governance within EAAA to include formalised QA of the reviewing clinicians’ reports by external POCUS experts. This additional external review will reduce the potential for bias and allows a final tier to ensure quality and maximise learning from clinical cases.

## Conclusion

In this service evaluation study, we have described outcomes following the introduction of a new POCUS device, image archiving system and governance framework in our HEMS. Paramedics were the primary scanning clinician in a quarter of scans, with image quality comparable to physicians. Almost all scans underwent quality assurance and inter-rater reliability was strong between clinicians and reviewers. Further research is required to investigate the diagnostic accuracy of POCUS and to demonstrate the effect of utilising prehospital POCUS to refine diagnosis on clinical outcomes.

## Data Availability

The datasets used and/or analysed during the current study are available from the corresponding author on reasonable request.

## Abbreviations

AAA: Assessment of the abdominal aorta
BMUS: British Medical Ultrasound Society
EAAA: East Anglian Air Ambulance
EEAST: East of England Ambulance Service
E-FAST: Extended focused assessment with sonography for trauma
ELS: Echocardiography in life support
EMS: Emergency Medical Services
FN: False negative
FP: False positive
FUSIC: Focused Ultrasound in Intensive Care
HEMS: Helicopter Emergency Medicine Service
IRR: Inter-rater reliability
LUS: Lung ultrasound
TN: True negative
TP: True positive
QA: Quality assurance
RCEM: Royal College of Emergency Medicine
RCR: Royal College of Radiologists
USG-VA: Ultrasound-guided vascular access
